# Assessing friction and damage of cell monolayers on soft substrates *in vitro*

**DOI:** 10.1098/rsif.2023.0696

**Published:** 2024-06-06

**Authors:** Rasmus Wagner, Matt J. Carré, Cecile M. Perrault, Paul C. Evans, Roger Lewis

**Affiliations:** ^1^ Department of Mechanical Engineering, University of Sheffield, Sheffield S1 3JD, UK; ^2^ Eden Microfluidics, Paris 75012, France; ^3^ Department of Infection, Immunity & Cardiovascular Disease, University of Sheffield, Sheffield S10 2RX, UK

**Keywords:** tribology, biotribology, medical devices, cardiovascular

## Abstract

In the area of surgical applications, understanding the interaction between medical device materials and tissue is important since this interaction may cause complications. The interaction often consists of a cell monolayer touching the medical device that can be mimicked *in vitro*. Prominent examples of this are contact lenses, where epithelial cells interact with the contact lens, or stents and catheters, which are in contact with endothelial cells. To investigate those interactions, in previous studies, expensive microtribometers were used to avoid pressures in the contact area far beyond physiologically relevant levels. Here, we aim to present a new methodology that is cost- and time-efficient, more accessible than those used previously and allows for the application of more realistic pressures, while permitting a quantification of the damage caused to the monolayer. For this, a soft polydimethylsiloxane is employed that better mimics the mechanical properties of blood vessels than materials used in other studies. Furthermore, a technique to account for misalignments within the experiment set-up is presented. This is carried out using the raw spatial and force data recorded by the tribometer and adjusting for misalignments. The methodology is demonstrated using an endothelial cell (human umbilical vein endothelial cells) monolayer.

## 1. Introduction

Some of the most popular cardiovascular medical devices (CMDs) are catheters, stents and stent retrievers. Stents are tube-shaped mesh structures used to open a blocked blood vessel. Catheters are used to deliver or retrieve fluids to or from various locations in the body. Stent retrievers are used to retrieve blood clots. All these devices touch the blood vessel wall during their application and operation, which causes mechanical interaction at the interface between endothelial cells (ECs) and medical devices. While stents are stationary, catheters and stent retrievers move relative to the vessel surface during their operation. Hence, for both groups of devices, friction plays a role, with dynamic friction being more important in catheters and stent retrievers, while static friction plays a major role for stents. Stents that do not apply enough static friction between stent struts and vessel walls can migrate owing to blood flow. Tribology has been successfully used in other areas to study the interaction between biomaterials such as tissue or bone and materials that have been introduced into the body in the context of implants, such as for artificial joints or hip replacements [1–3].

The applications of CMDs come with risks of complications, some of which can be linked to mechanical trauma or damage to the endothelium. In the physiological context, the damage caused by frictional interaction refers to trauma caused to the endothelial layer. As cell monolayers are not a conventional material in tribology, specific methods are required to make the damage or ‘wear’ visible and to quantify it in this context.

*In vitro* friction experiments have been conducted on (endothelial) cells before [4–8]. The overwhelming majority of those studies were carried out using cells cultured on either flat samples of polystyrene (PS) or glass, both of which are very good materials for cell culture. However, they are extremely hard compared with *in vivo* materials. Some of those studies have used soft (hydrogel) counter surfaces to avoid high pressures; however, since hydrogels only represent a realistic medical device material for a limited number of applications (contact lenses and some catheters), many studies have used glass or stainless steel spheres as the contacting ‘probe’ for testing. This makes the experiments reproducible and comparable, as accurately manufactured spheres of these materials can be procured anywhere in the world with relative ease. Nevertheless, these material pairings mean that the cells are stuck between a hard substrate and a hard sphere. Unless expensive microtribometers are available enabling extremely low loads to facilitate the application of low contact pressures, this results in almost all the cells in the contact area being destroyed owing to extreme compression, which makes damage difficult to assess, produces conditions that are not realistic and leads to the total obliteration of the cells. Those conditions also raise the question of the quality of the lubricating layer: it is not clear whether an intact endothelial monolayer or mangled cell remains are tested. The methodology to create soft substrate samples with a monolayer of human umbilical vein endothelial cells (HUVECs) shown in this work was developed with this in mind and was used to test cells in a more realistic way that also makes it possible to assess damage. In contrast, the exact same methodology (except for the soft substrate) was applied to stiff PS-based samples in order to see how the soft substrate influences the results.

This study aims to further improve tribological experiments on cell monolayers by mimicking the mechanical properties of blood vessels better than previous studies using a soft substrate. A live/dead assay is used to determine cell damage. Furthermore, a method is proposed to identify and account for misalignments based on the raw position and force data output by the tribometer. Not many studies share information on how raw data were processed and where friction forces were measured. The method presented in this work allowed us to study the effect of varying normal force on damage and friction. It also enables research groups that cannot afford a specialized biotribometer but have access to a generic tribometer capable of measuring low loads to conduct experiments on cell monolayers.

This work aims to provide a framework to mimic the frictional interaction between blood vessels and medical devices in an *in vitro* environment under more realistic conditions. For this, glass spheres were tested against a blood vessel substitute consisting of ECs, fibronectin (FN) and a soft polydimethylsiloxane (PDMS) to study the influence of load parameters. Future work may explore the relationship between probe material properties as an input and friction and damage as an output.

## 2. Background

**Figure 1.**
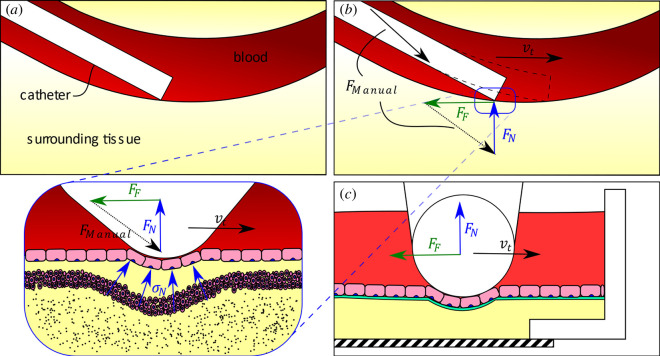
Catheter sliding on blood vessel (*a*,*b*) and *in vitro* replication (*c*) with tangential speed $v_{t}$ under normal force $F_{N}$ , resulting in friction force $F_{F}$ . In the *in vivo* situation, $F_{N}$ and $F_{F}$ result in a total force $F_{manual}$ that must be overcome by the surgeon.

The interaction of CMDs, specifically stents and catheters, against blood vessels is not well understood until now. Reliable friction data could provide more accurate simulations and models. Understanding the endothelium’s reaction to frictional interaction with medical devices and the resulting damage can inform new medical device designs. This would result in making an intervention safer and less invasive by reducing complications such as the risk of traumata and de-endothelization, with possibly following atherosclerosis. Additionally, when using catheter intervention, lower friction could mean less resistive forces and, therefore, a more pleasant and overall smoother process for the patient [9]. The interaction of a catheter with a blood vessel and the respective *in vitro* replication is shown schematically in figure 1.

New materials need to be tested to achieve the goal of making cardiovascular intervention safer. Generally, the relevancy of the experiment to real applications increases with increasing complexity. However, cost and timeframes become bigger for such tests, and for *in vivo* experiments and medical studies, animals are often used, which is undesirable. Therefore, it is important to test new materials using *in vitro* experiments under laboratory conditions first before advancing to *ex vivo* and then *in vivo* testing, gradually increasing complexity and ensuring that new materials are tested extensively before their application in animals and, finally, humans. Ideally, we would want *in vitro* tests to be representative enough that human/animal testing is not needed; however, at the moment, *in vitro* experiments alone are not capable of serving as a sufficient substitute.

As such, *in vitro* experiments are an important step in the development process as they come with the advantages of controlled laboratory conditions while providing results collected from living tissue. They also allow an easy investigation by means of fluorescence and light microscopy. For these reasons, they can be relatively cheap to conduct, quick to set up and, at the same time, accurately emulate real tissue. As the experiment was designed to investigate the interaction between medical devices and blood vessels, the methodology presented will involve HUVECs. However, it could easily be adapted to mimic different tissue types, such as cornea or urinary tract tissues by varying the cell type.

## 3. Experiment set-up and protocols

As a baseline, friction experiments were conducted with FN-coated PS dishes (PS + FN). Friction tests with cells were conducted on the same type of dish cultured with cells, which represents the current state of the art for most cell friction experiments (PS + FN + HUVEC). Furthermore, experiments were conducted on a soft substrate sample using soft PDMS (PDMS + FN + HUVEC). An overview of the testing conditions and repeats can be seen in table 1. Experiments with cells were repeated nine times because cells represent an additional variable that cannot be easily controlled. Furthermore, because the cell density was measured after testing for these experiments, a higher number of repeats was chosen than for the ones without cells. Testing PDMS with and without FN showed that friction forces exceeded the limits of the load cell set by the manufacturer and also caused the PDMS to rip. This type of damage was not observed when cells were tested on PDMS, likely owing to the much lower friction forces occurring.

### 3.1. Samples

The sample, in the scope of this experiment, means everything that represents the biological tissue. Two critical requirements were identified for the sample.

First, it must have a surface that is as close to that of a real artery as possible. This is because adhesion effects between cells and medical device surface that could be caused by cellular membrane molecules may play an important role. Additionally, to assess damage to the endothelium, the behaviour of the surface must be similar. A HUVEC monolayer was chosen to replicate *in vivo* surface properties.

Second, it should replicate realistic mechanical properties, which not many other studies have considered. This means that the substrate underlying the cell monolayer should be soft for cardiovascular applications rather than hard like glass or polystyrene substrates. This property is important as pressures are more realistic if a soft substrate is used. The material can deform, just as a real artery (or another human soft tissue such as eyes) would. This avoids introduction of unrealistic pressures in the cells that would be stuck between two hard bodies (probe and glass or polystyrene substrate). For other applications, for example if the underlying real tissue is bone, a hard substrate may be the correct choice, as it could replicate realistic conditions.

**Figure 2.**
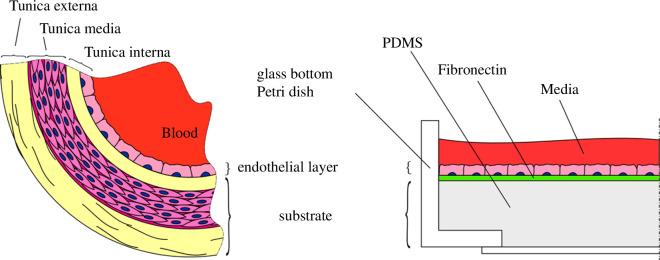
Structure of an artery and of PDMS-based sample used for *in vitro* experiments in comparison.

The soft sample structure is shown in figure 2. Tunica media and tunica externa were represented by a soft PDMS. FN was used to attach the cells to the substrate. HUVECs were seeded on the substrate.

#### 3.1.1. Substrate

Standard 35 mm culture dishes with a glass bottom (CELLView™, Greiner Bio-One, item no. 627860) were selected to hold the sample, as the glass bottom allows a clearer view of the cells than a regular polystyrene dish would.

As a substrate, soft PDMS (NuSil, GEL-8100) with 1% additional crosslinker (Sylgard 184 curing agent from DOWSIL formerly DOW CORNING) was prepared according to the protocol of Yoshie *et al*. [10]. This substrate has a Young’s modulus of 73.32 ± 2.96 kPa [10]. Each dish was filled with 2 g PDMS, which is equivalent to approximately 2 ml. Given that the area of one culture dish is *ca* 8.8 cm^2^, the approximate height of PDMS is 2.27 mm. For some studies, a harder substrate might be desirable. This could be the case if ECs growing on a calcified artery are to be studied or, for example, in cartilage-focused research. Sylgard 184 is a harder PDMS that could be used for this.

The PDMS was cured at 65°C for 8 h (longer than the 90 min specified by Yoshie *et al*. [10]). This was to ensure thorough curing of the polymer without repercussions because the product is stable at up to 240°C.

After curing, the dishes were sterilized by fully submerging them in a larger cell culture dish filled with 70% IMS and left in the cell culture hood for 30 min. Then, the dishes were transferred to another larger cell culture dish, fully submerged with phosphate-buffered saline (PBS), washed by swilling gently and left for 30 min. Washing with PBS was repeated three times in total.

#### 3.1.2. Coating

To make the substrate more accommodating for cells, the PDMS was coated with 1 μg cm^−2^ (1−5 μg cm^−2^ is recommended by Sigma for ECs; this value might differ for other cell types) of FN (Merck, cat. no. F1141). Cells can attach to FN because they have cell adhesion molecules able to adhere to the protein that is naturally found in the extracellular matrix. The appropriate amount of FN (8.8 μg per dish) was diluted in 3.5 ml PBS per dish as this volume was sufficient to cover the whole culture dish surface. The solution was mixed with a pipette and each dish was filled with 3.5 ml solution. The dishes were left in a cell culture hood at room temperature overnight. Alternatively, the substrate could be coated with another protein or gelatin for different cell types. When the coating process of the substrate was finished, the sample was washed three times with PBS.

#### 3.1.3. Cell culture

HUVECs (catalogue number C-12253) were acquired from Promocell, Germany and cultured using the appropriate culture medium (catalogue number C-22010) in T25 and T75 flasks. When the cells in the flask were about 90–100% confluent, the cells were ready for detaching with trypsin (an enzyme that dissolves the extracellular matrix) and seeding on samples. As cells were cultured in regular culture dishes, and friction tests were conducted in a non-sterile environment, sterility could not be fully guaranteed. Hence, the culture dishes could be infected by bacteria or fungi. To avoid infections, cell culture was conducted with penicillin and streptomycin added to the culture medium.

After trypsinization, the detached cells were collected with a pipette. After centrifuging, the cell pellet was diluted in a culture medium, and the cells were counted with a haemocytometer. Cells were seeded with a density of 10 000 cells cm^−2^, resulting in 88 000 cells per dish, diluted in a 3.5 ml cell culture medium. The samples containing cells were stored in an incubator at 37°C, 95% relative humidity and 5% CO_2_. The culture medium was changed every 2–3 days until confluency was reached (after 7–10 days), and as such, the sample was ready for tribological testing. The cells were tested deliberately at full confluency and not after a fixed time as the target was for the sample to represent the usual state of an artery *in vivo*, that is, a confluent monolayer.

The procedure specified in this section is standard cell culture protocol. A similar approach could be used for different cell types such as epithelial cells, allowing for studying urinary catheter and contact lens interaction in their respective environments, requiring a different culture medium and similar incubation conditions.

### 3.2. Probe

The probe replicates the medical device surface and geometry. To obtain realistic results, it is important to use a material that is similar to that of a real device. For catheters, that would be polyurethane, stents would be represented by stainless steel probes and contact lenses could be represented by a hydrogel probe as shown by Marshall *et al*. [11]. In the scope of this article, 2 mm diameter soda lime glass probes from Atlas Ball & Bearing (ABB), UK were used. Glass does not represent a realistic medical device, but it has been used many times in the past for biotribological experiments. Since this article focuses on presenting a methodology to conduct experiments, the results should be comparable to those of past studies. Hence, glass was chosen as the probe material. Furthermore, glass probes are readily available and, therefore, provide a good baseline despite not necessarily delivering medically relevant results. For the probe geometry, a sphere was chosen as it is a frequently used shape for friction experiments featuring a defined and analytically calculable contact geometry that is important for repeatability and calculation of pressures.

**Figure 3.**
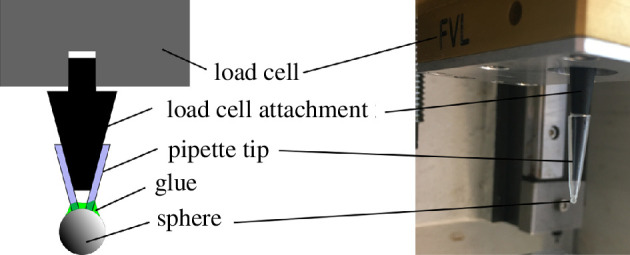
Schematic view of probe geometry and photo of the probe in the UMT.

A photo and a schematic of the probe set-up are shown in figure 3. The spheres were glued to pipette tips with cyanoacrylate glue. The load cell attachment is a three-dimensionally printed geometry that serves as a connector between the load cell and the pipette tip with the sphere. As the pipette tip can be pushed over the load cell attachment, a quick swap of probes between friction experiments was possible. This is important as three slides were tested per sample and time spent outside the incubator should be minimized.

### 3.3. Testing

**Figure 4.**
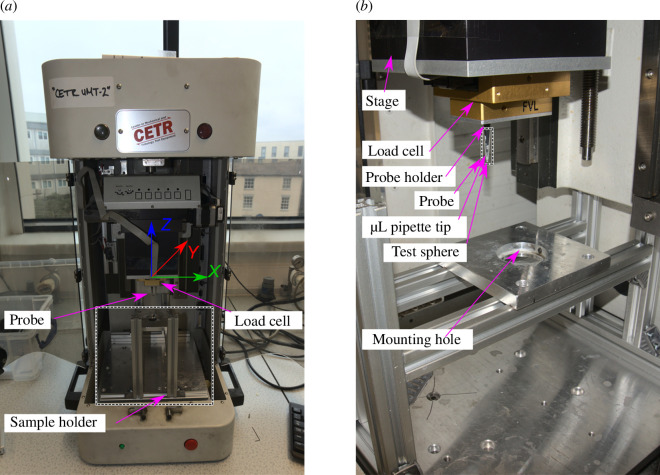
(*a*) UMT2 with custom sample holder and main parts labelled. The coordinate system with the *x*, *y* and *z* axes is also shown. (*b*) FVL load cell with attached probe and sample holder.

In this experiment, a pin-on-plate set-up was used to apply a single slide over the substrate. The normal load was set to a fixed value. Testing was conducted in a UMT2 from Bruker. For the measurement of forces in *x* and *z* directions, a Bruker FVL load cell was used, which features a maximum load of 100 mN with a resolution of 1 µN. The UMT2 and the FVL load cell are shown in figure 4.

Friction coefficients between ECs and glass can be expected to be around 0.03–0.06 [5]. Therefore, a tribometer capable of measuring low loads is required. The tribometer can move the probe in two directions, normally and tangentially to the sample surface. For the following experiments, the tangential speed was kept constant at 1 mm s^−1^ but could be set up to 3.2 mm s^−1^ in future experiments with the available machine. The tribometer also measures forces in the two directions of movement. It can control the load by lifting or pressing down the load cell with the probe on the substrate by moving the stage in the vertical direction. For the UMT, the vertical speed can be specified using three parameters: pre-touch, touch and tracking. Correct specification of these parameters can be crucial since poor choice may cause the normal load to oscillate, the UMT to call a timeout error or the normal force to diverge from its set value. The values chosen for the two substrate materials in this work can be found in table 2. Values were found through trial and error. When the velocity was set too high, the machine could overshoot the applied force and oscillate or even abort the test. If set too low, the machine would not be able to track the slope of the surface, and, hence, the normal force may diverge.

**Table 1.** Experiment overview.

substrate material	countersurface (probe) material	relative speed	repeats for each load condition
PS + FN	glass	1 mm s^−1^	3
PS + FN + HUVEC	glass	1 mm s^−1^	9
PDMS + FN + HUVEC	glass	1 mm s^−1^	9

**Table 2.** UMT engage and tracking settings used for experiments on PS- and PDMS-based samples.

parameter [unit]	polystyrene (PS)	polydimethylsiloxane (PDMS)
vPretouch [mm s^−1^]	0.15	0.5
vTouch [mm s^−1^]	0.03	0.15
vTracking [mm s^−1^]	0.01	0.1
FN,Touch [mN]	−1	−0.3

The sample holder was levelled with respect to the axis using an empty culture dish and ensuring that the test surface was flat. Samples were levelled during curing with a digital spirit level. However, one of the main advantages of this methodology is that certain inconsistencies during levelling may be mitigated owing to the curvature of the sample.

Cells need specific environmental properties to survive. Human cells are usually cultured at 37°C, with 5% CO_2_ and with 95% relative humidity. Owing to the short nature of the experiments described in this article (less than 10 min testing time), none of these properties was controlled during testing. As the sample cannot dry out, the humidity does not have to be controlled. Also, the short testing time does not allow for the pH to change significantly, which is the main purpose of controlling CO_2_ content. As the cells were cultured on plastic and submerged in warm media, the temperature was not assumed to change significantly either. If this methodology was applied for long-term experiments, the tribometer would have to be capable of maintaining the temperature of the sample, pH value of the media and humidity. The best option to maintain these properties is a small environmental chamber. If this option is not feasible, the temperature could be controlled using a culture dish heater. To control the pH value of the media, a buffer, such as HEPES buffer, could be used. High humidity is necessary to maintain the right concentration of nutrients in the media as concentrations rise if water evaporates. This effect should only come into play for very long experiments and could be mitigated by partial media changes during the experiment.

**Figure 5.**
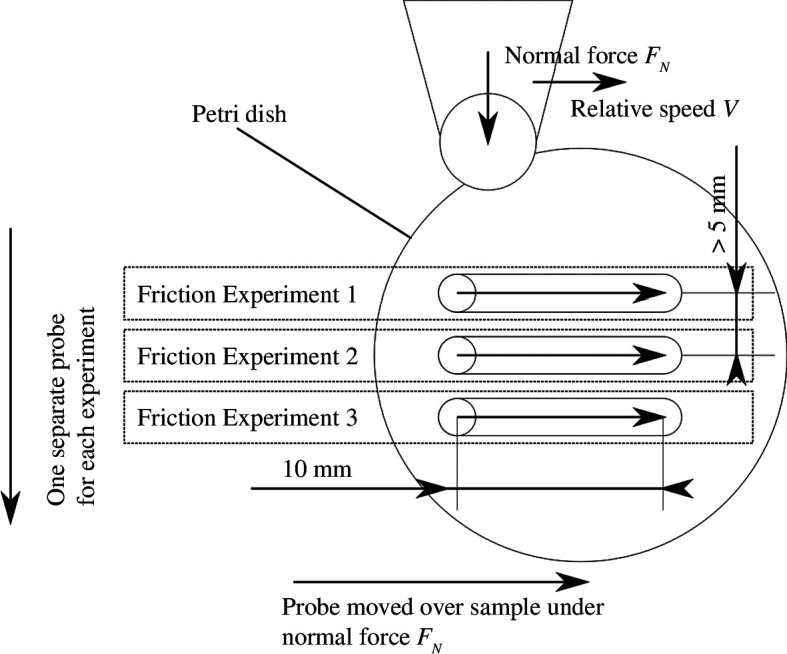
Test layout on the sample for friction experiments.

The sample was tested at several locations to maximize efficiency and cost-effectiveness as per the layout shown in figure 5. One sample was large enough to conduct three friction experiments on it. Only a single slide in one direction over the monolayer was studied to limit complexity. Future studies could look at reciprocating load conditions.

### 3.4. Staining and imaging

After testing, the cells may be imaged with the objective of measuring cell damage and cellular reactions, which is called a live–dead assay. In general, live–dead assays are based on two markers. The first marker can enter all cells, including those with a healthy cell membrane. The second one is only able to enter cells with a dysfunctional (damaged) cell membrane owing to the size of the molecule. More advanced techniques may include staining for inflammatory or apoptotic markers.

An easy and readily available method to assess cell damage is trypan blue. Trypan blue cannot permeate a functioning cell wall. This allows cells that suffered damage to be stained blue, which can be detected under a regular light microscope. The stain was tested and showed good results; however, without a counter stain, it is difficult to distinguish areas populated by living cells from areas where cells were removed.

To solve this issue, the cells were stained with propidium iodide (PI) from Sigma-Aldrich (catalogue number P4170) to detect damaged cell nuclei. Additionally, Hoechst 33342 (Thermo Fisher Scientific, H3570) was used as a counterstain to distinguish between areas with live cells and areas without cells remaining. Like trypan blue, PI can only enter cells with a dysfunctional or ruptured cell wall, and it stains for DNA. Unlike trypan blue, PI is only visible under a fluorescence microscope and shows up in the red channel (excitation: 488 nm; emission: 617 nm). As a counterstain for undamaged cells, Hoechst, which can enter live cells and stains for DNA, was used. Hoechst is visible in the blue channel of a fluorescence microscope (excitation: 361 nm; emission: 486 nm). For imaging, a Nikon Eclipse Ti with a CoolLED pE-300 for fluorescence imaging was used. Both stains were applied as a single solution containing 0.15 mg ml^−1^ PI and 1 µg ml^−1^ Hoechst in a PBS buffer.

With these markers, there are three states a cell can show up after testing that are relevant regarding damage. First, the cell can have survived and be attached to the substrate. Such a cell would count as undamaged in the context of this analysis. Second, the cell could still be attached but have suffered a rupture of the cell membrane, eventually leading to cell death. Third, a cell could have been completely detached (either dead or alive). The latter two count towards cells that did not survive the interaction.

After tribologically testing the samples, the medium was aspirated and the sample was washed with 2 ml PBS, and 1 ml of pre-warmed (37°C) staining solution was added to the sample. After incubating for 15 min, the staining solution was aspirated, and the sample was washed with 2 ml PBS and then filled with 1 ml PBS. Afterwards, the sample was imaged under a fluorescence microscope. The cells were not fixed at this point since this was found to adversely affect staining quality, so the imaging process was conducted immediately after staining.

Imaging of the monolayer was not practical before testing since a large area had to be imaged, and quick processing of the sample containing living cells was required. Furthermore, staining was only conducted after testing so as to not induce more stress in the monolayer. With an improved set-up that would allow for imaging below or in front of the probe, capturing brightfield images before testing could be realized.

### 3.5. Data analysis

#### 3.5.1. Friction data

**Figure 6.**
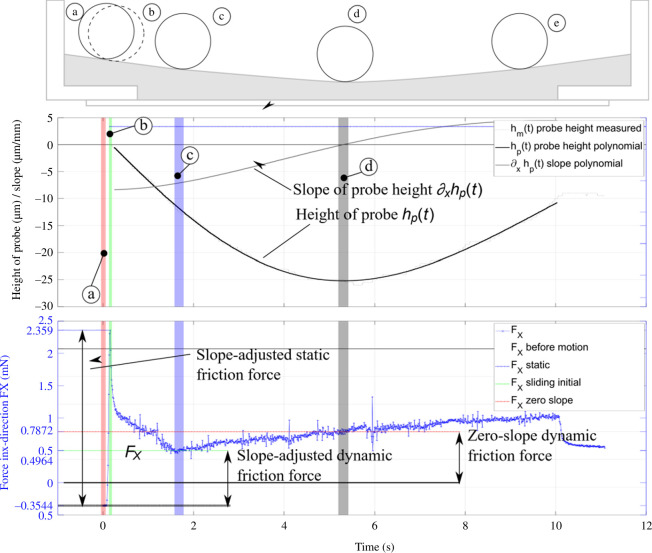
UMT friction force FX (blue), height of probe *h* (black) and slope $\partial_{x}h$ (grey) plotted over time with probe position illustrations. Different stages are marked by coloured boxes: (*a*) (red)—pre-movement; (*b*) (green)—static friction; (*c*) (blue)—friction force stabilized; (*d*) (grey)—friction force at zero slope; (*e*) (only on probe position illustration)—sample is moving up the slope; hence, the friction force gets bigger.

During analysis of the friction data, it was apparent that the height of the probe was not consistent over the sample. It turned out that the probe both moved on a slope and also followed a meniscus shape. The first is caused by the misalignment of the sample with the stage. The latter is an issue of the methodology as the polymer is cured from a fluid base that may cause a changing slope over the sliding distance owing to meniscus effects or could be caused by stresses introduced during curing. If using polystyrene dishes without a soft substrate, for example, the meniscus may not be visible, but the misalignment between stage and sample may still influence the results. Misalignment and meniscus must be considered; however, the meniscus was found advantageous as it sometimes gave a level section at least somewhere along the sliding distance. The effect is shown in figure 6 and overlaid with experimental data. A method to account for misalignments based on a mechanical model developed within the context of this work is presented in appendix A.

#### 3.5.2. Cell damage

To analyse the damage caused to the cell monolayer, a workflow was created that uses the live/dead assay (Hoechst/PI). Cells were stained with PI which stains damaged cells. Additionally, the sample was stained with Hoechst 33343 which stained the remaining cells. Since the two dyes have different colours under the fluorescent microscope, damaged and healthy cells could be distinguished.

## 4. Results

### 4.1. Damage inflicted to the monolayer

**Figure 7.**
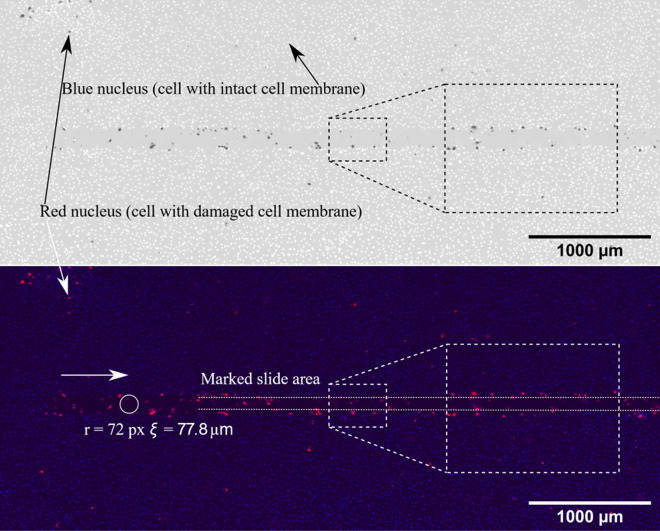
Red/blue combined image of a 10 mN normal force slide. The slide direction is marked by a white arrow. Top: inverted black and white image with contrast boost. Blue nuclei are white and red ones are dark in black and white image. Bottom: colour image with original red/blue channels.

**Figure 8.**
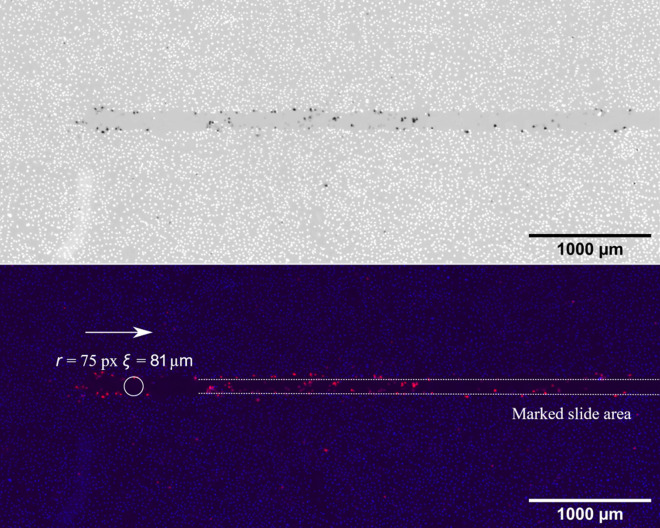
Redx/blue combined image of an 80 mN normal force slide.

In figure 7, a 10 mN normal force slide is shown. Even for small forces, the monolayer was destroyed within the slide track, and almost no cells remain attached to the track. When the normal force was increased to 80 mN, the width of the slide track did not increase significantly, as shown in figure 8. The width of the track was measured as indicated by the circles. For a normal force *F_N_* of 10 mN, the average width *r* was approximately 77.8 µm and for *F*_*N*_ of 80 mN around 81 µm. However, it should be noted that the width could vary along the track. A detailed description of how the monolayer damage was calculated can be found in appendix B.

**Figure 9.**
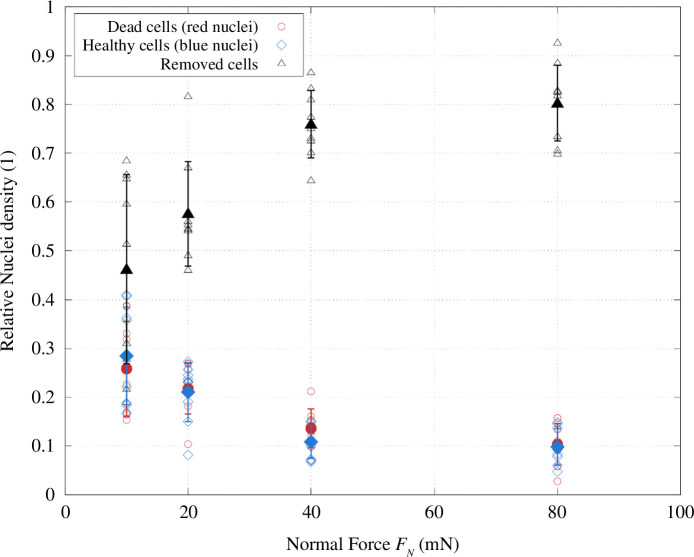
Damage caused to HUVEC monolayer on PS by the glass probe. Nuclei densities in the slide area relative to the blue nuclei density in the respective reference areas P are plotted over *F_N_*. Measurements of one slide are plotted as hollow circles, while averages are represented by filled circles. Blue circles stand for blue density measurements, red circles for red ones and black circles for the calculated percentage of removed cells.

Apart from estimating the width of the track, the slide images that were collected were used to study the damage caused to the monolayer. On average, for normal forces of 10 mN, 28% of cells remained healthy in the slide area, while 26% were dead. Overall, 46% of all cells were removed in the slide area. When the normal load was increased to 20 mN, 21% of healthy cells remained, with 22% dead and 58% removed. Increasing the normal force further to 40 mN resulted in 11% of healthy cells remaining and 14% dead ones with 76% of cells removed. Finally, an increase in *F_N_* to 80 mN prompted the most fatal damage to the monolayer that was recorded with 10% healthy cells and also 10% dead cells in the slide area. In total, 80% of the cells within the slide area were removed in the friction process on average. These data are plotted in figure 9 for the range of normal forces by showing the relative densities of nuclei in the test area with respect to the reference areas.

**Figure 10.**
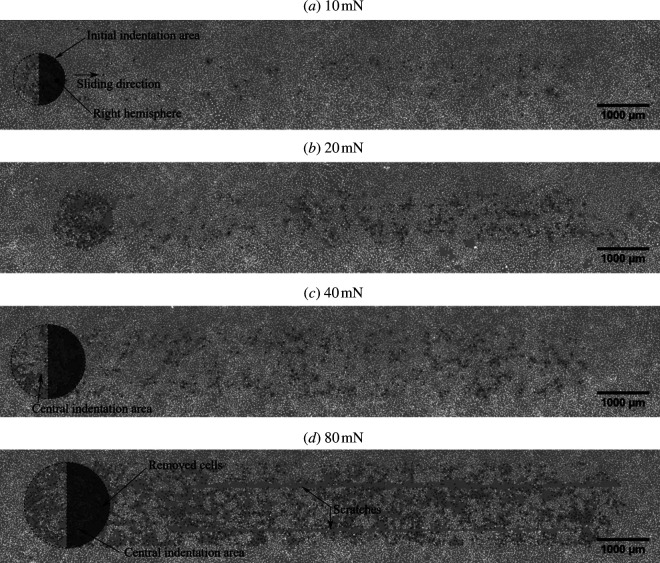
Slide track images of soft substrate samples tested against glass probes for *F_N_* of 10, 20, 40 and 80 mN.

In figure 10, representative images of the track are shown for the full range of forces on soft substrates. The circular initial indentation area and its right hemisphere are marked. The right hemisphere is the one facing towards the sliding direction. The sliding direction is indicated in the first slide image and is the same in all other images. As a general trend, in the right hemisphere, the damage to the monolayer was noticeably higher. For normal forces of 10 and 20 mN, this resulted in more cells found dead in that region and for higher normal forces of 40 and 80 mN rather than more dead cells being present, more cells were removed there. For a normal force of 80 mN, scratches in the monolayer become apparent, which are highlighted in the respective image.

**Figure 11.**
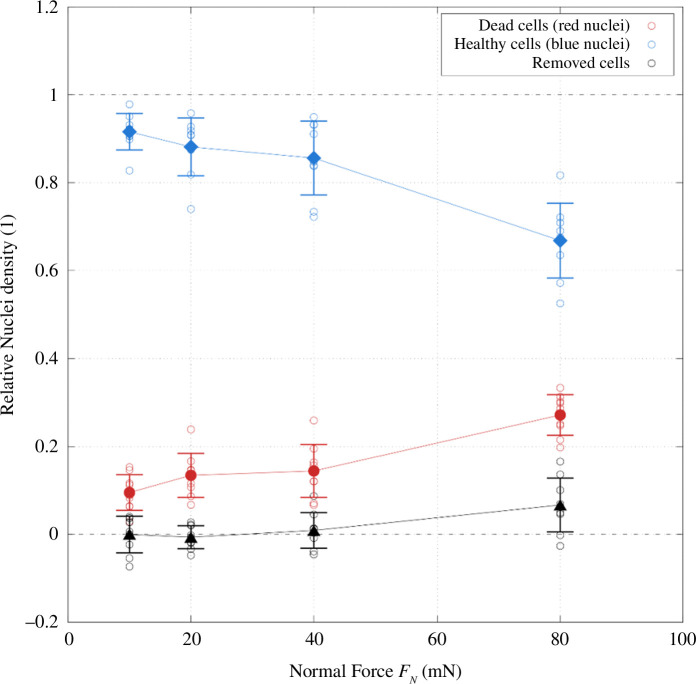
Monolayer damage data for PDMS-based samples with HUVECs tested against glass probes presented as relative nuclei densities with respect to the compared areas. Dead cell densities are plotted in red, densities of healthy nuclei in blue and calculated removed densities in black. Individual measurements are plotted as open symbols, while averages are represented by filled symbols with standard deviation.

The resulting damage caused to the monolayer by the interaction of the glass probes with the soft substrate samples is shown in figure 11 for different loads. Singular slide measurements are plotted as open symbols and averages as larger, filled ones with the respective standard deviation. Blue nuclei densities are represented by blue symbols, red by red symbols and the calculated removed densities by black symbols.

For 10 mN normal force, barely any cells were removed from the sample. So few cells were removed that for some slide areas, measurements of removed cell densities are below 0, which means that there were more cells in the slide area per unit area than in the reference areas, implying that cells were added in the process of sliding. This was likely owing to natural variances in the cell density of the monolayer. On average, −0.04% of cells were removed. 9.52% of cells were dead in the slide area, with respect to the density of healthy cells in the reference areas, and 91.57% were healthy. This means that essentially no cells were removed, and the damage is limited to individual cells being killed. The values for red and blue relative nuclei densities do not add up to 100% despite barely any cells being removed, which is again likely owing to variances in the initial nuclei density and the fact that relative nuclei densities are based on the blue nuclei density in the respective reference areas. For a normal force of 20 mN, still no significant amount of cells (−0.64%) was removed. However, more cells (13.44%) were dead for this increased normal load, which was naturally accompanied by a reduction in the rate of healthy cells in the slide area (88.12%). An increase in *F_N_* to 40 mN caused 0.90% of cells to be removed. The rate of dead cells increased slightly further to 14.43%, while the amount of healthy nuclei decreased to 85.58%. Overall, the damage inflicted on the monolayer increased only slightly from 20 to 40 mN normal force. Finally, for a normal force of 80 mN, the amount of removed cells became significant as it increased to 6.70%. Also, the amount of dead nuclei increased further to 27.19%, while only 66.78% of cells remained in the slide area.

### 4.2. Friction

PS dishes were coated with FN and seeded with HUVECs (PS + FN + HUVEC). Furthermore, PS dishes were coated with FN and tested without seeding cells on them (PS + FN). Dishes were then tested in the UMT2 against glass probes under the same conditions and with the same procedure used for soft substrate samples.

**Figure 12.**
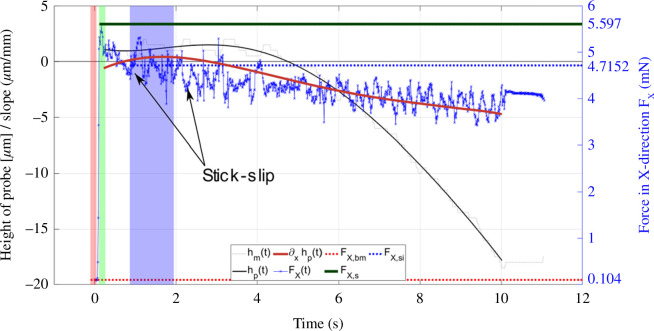
Raw friction force $F_{x}$ and measured stage position $h_{m}$ data for a 10 mN friction test on a PS-based, FN-coated sample. Additionally, the height is approximated by a polynomial of fifth order $h_{p}$ and its slope $\partial_{x}h_{p}$ are plotted. Values for force in the *x* direction before the experiment begin $F_{x,bm}$ , static friction force $F_{x,s}$ and friction force after movement began $F_{x,si}$ are highlighted.

**Figure 13.**
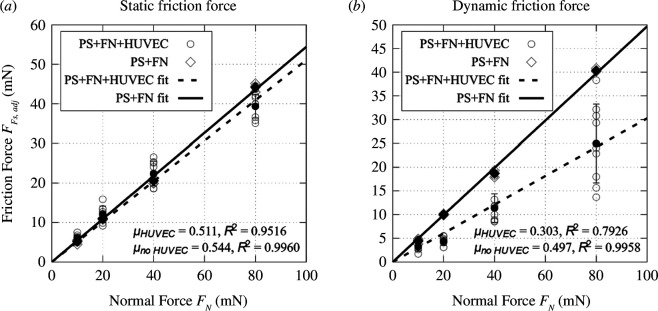
Static (*a*) and dynamic (*b*) friction of glass against FN-coated PS with and without HUVECs. Slope-adjusted static friction force *F*_Fs,adj_ and slope-adjusted dynamic friction force *F*_Fd,adj_ plotted over applied normal force *F_N_*. Individual measurements and averages with standard deviation are plotted in addition to a linear fit as per Amonton’s law; FF = *µF_N_* for both materials.

Static and dynamic friction forces of glass probes on PS + FN + HUVEC and PS + FN were extracted from the friction data as described earlier. The raw data for a 10 mN experiment on PS + FN are shown in figure 12. A noticeable degree of stick–slip was observed. In figure 13*a,b*, static and dynamic friction forces are plotted over the whole range of tested normal forces.

For the static friction, the value for the friction coefficient *µ*_s,HUVEC_ = 0.511, as fitted with the linear model Amonton’s law suggests, is smaller than the one of just FN-coated PS (*µ*_s,PS + FN_ = 0.544). For the dynamic condition, the friction coefficient *µ*_d,HUVEC_ = 0.303 was significantly smaller than the one on the same substrate without cells (*µ*_d,PS + FN_ = 0.497) and the one on just PS (*µ*_d,PS_ = 0.474). It should be noted that especially for dynamic friction, the spread of the measured values is higher than it is when no cells are present. However, the highest measurement for the dynamic friction force is just below the ones of FN-coated PS. More friction results may be found in appendix C.

**Figure 14.**
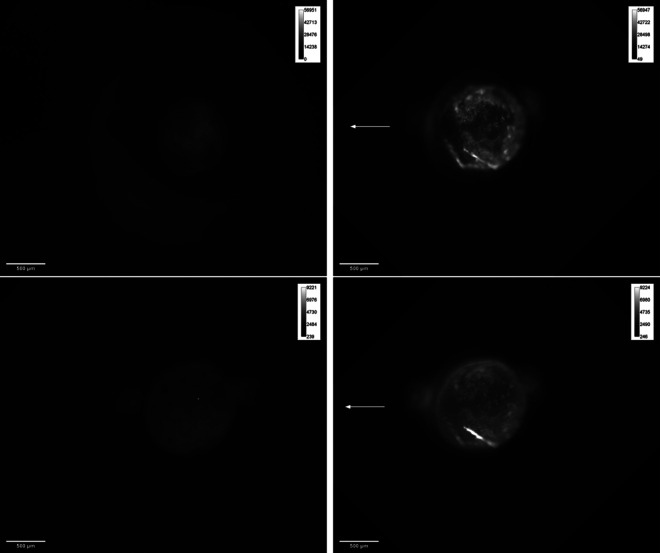
Images of a glass probe after sliding over the monolayer with a normal force of 10 mN. Left: glass probe before testing. Right: after testing, stained with dapi and phalloidin. Top: blue channel images (dapi). Bottom: red channel images (phalloidin).

It should be noted that a cell remaining adhering to the probe may influence friction or adhesion between the sample and the probe. Figure 14 shows a glass probe before and after testing. Cell remains were clearly visible in fluorescence channels, showing that cell remains adhered to the probe.

## 5. Discussion

For standard PS dishes with HUVECs, it was observed that the slide track was significantly wider than the Hertzian contact theory implies for a PS–glass contact. Furthermore, the width of the track did not increase to the extent suggested by Hertz with an increase in the normal force. The magnitude of the difference between the data and the Hertzian calculation is beyond the effects expected owing to limitations of the model such as adhesion, plastic deformation and nonlinear material parameters. It should be noted that the width of the track was measured based on damaged and removed cells, and in the Hertzian calculation, it was assumed that the cell layer was not playing a major role. From a purely mechanical standpoint, the assumption seems fair. However, the fact that cells are mechanically fragile compared with the other materials involved in the interaction may help to explain what is going on, focusing on the issues of deviations to the contact theory model and, consecutively, with the one of a minimal change in contact width with an increase in the normal force.

In the context of this work, a model was created based on Hertzian contact mechanics that explains the small change in the contact width with an increase in the normal force observed here. This model was also tested against previous work and was able to predict track widths there. Essentially, the phenomenon can be explained by the combination of two effects. First, the cell monolayer is soft and easily pierced, so the cells in a certain radius around the centre of the indentation are destroyed. This radius depends on the diameter of the probe. Once the cell monolayer is pierced, the PS absorbs the majority of the normal load, and since it has a fairly high Young’s modulus, deformations are small, causing the track width to only vary slightly with the normal force. The model is explained in more detail and applied to previous work in appendix D.

With regards to friction on cells cultured on PS, the question about the nature of the contact may be raised: are cells being tested or are they merely a lubricant between the PS dish and the glass sphere?

In figure 13*a*, the static friction data for PS + FN and PS + FN + HUVEC samples are plotted. Comparing the data in the figure indicates that cells do not have a major influence on the static friction force. This is also reflected by the static friction coefficients 0.544 and 0.511 for PS + FN and PS + FN + HUVEC, respectively. The friction force is slightly reduced. However, it is still much higher than the static friction force measured using soft substrate samples, where the pressures were much lower, and, hence, significantly more cells survived and remained in the track. As shown earlier, the static friction does not behave according to Amonton’s law for soft substrate samples, but for reference, the fitted static friction coefficient was 0.053. Also, on soft substrate samples, the qualitative friction force behaviour as a function of normal load is very different. Both PS-based samples’ results were in reasonably good accordance with Amonton’s law of friction, while the results using the soft substrate samples were not.

For the dynamic load condition, the difference between the PS + FN and the PS + FN + HUVEC samples was more significant, as is evident from the fitted dynamic friction coefficients 0.497 and 0.303, respectively. Despite being smaller, the values for PS + FN + HUVEC stand in stark contrast with the dynamic friction coefficient fitted from measurements on soft substrate samples, 0.018. The latter stands in much better accordance with literature values (Dunn *et al*. report a friction coefficient of *µ* = 0.03–0.06 [5]).

**Figure 15.**
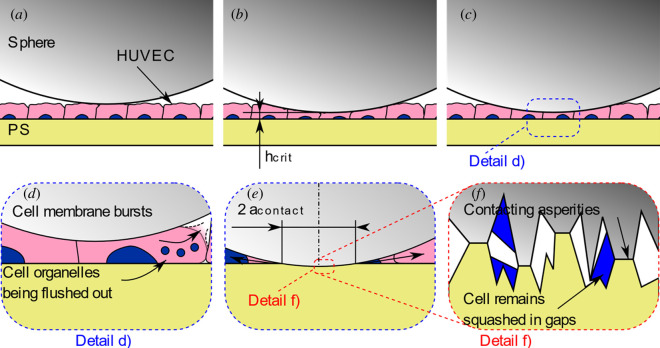
Schematic of the interaction between a glass sphere and a PS sample with HUVECs.

Figure 15 shows a possible reason for the small difference between PS + FN and PS + FN + HUVEC. The indentation process is shown at different stages. The sphere is shown as it just contacts the monolayer in position (*a*). In position (*b*), the cells in the centre of the contact are compressed to *h*_crit_ and are thus about to burst. The probe is lowered further in position (*c*), destroying the cells and squeezing out their contents. Detail (*d*) shows the bursting. As the pressure gradient is directed outwards from the contact area, the cell bursts at the side. Cells mainly contain cytoplasm that is largely made up of water, so a good analogy is a balloon filled with water that is squeezed. It will burst somewhere at the side of the cell facing away from the centre of the contact area (i.e. it will burst in the direction of the pressure gradient). When that happens to the cells, most of the contents, like cell organelles, will be flushed out with the cytoplasm. As the sphere moves further down until it is in contact with the PS (i.e. the probe is fully loaded with the PS taking the main load), all liquid parts of the cell will be squeezed out and only viscous and elastic parts can remain. Those are mainly parts of the cell that were in some way attached to the substrate, such as the nucleus, parts of the cytoskeleton and the cell membrane, which are bound to the substrate through adherens junctions. At this point, an area will have formed where the glass and the PS are very close. This area will be approximately circular owing to the symmetrical geometry with the radius $a_{contact}$ . Detail (*f*) shows this area on a microscopic level. The asperities of the glass and the PS may contact each other, as those materials are very hard compared with the cell organelles, and the pressures between the asperities can be expected to be very high. This would mean that in the static friction case, which has been preceded by 10 s of loading, the friction behaviour is largely equivalent to PS against glass, with some cell parts trapped in the asperities. This could be the reason why barely any difference was measured between PS + FN and PS + FN + HUVEC.

When the probe starts moving, more and more cell material comes into the contact area. As it is continuously moving, there may not be enough time for the probe to squeeze the more viscous cell parts out of the contact. Also, it is possible that at some point, the asperities of the sphere are saturated with cell matter. Both could lead to a separation of the asperities on a microscopic level as the cell matter is squashed between them. This would mean changing the dynamic of the contact mechanics and thus could be the reason for the observed reduction in the friction coefficient. In this case, the cells would act as a lubricant rather than as a surface in the interaction. This is supported by the observation that only 28.5% of the cells in the monolayer remain healthy, even for the lowest load tested, on PS samples.

**Figure 16.**
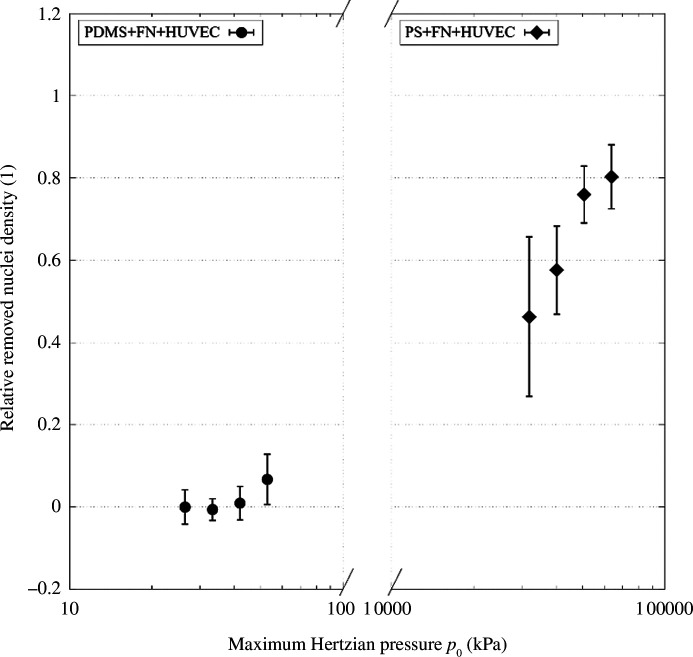
Removed nuclei data on PDMS-based and PS-based samples against glass probes over Hertzian contact pressure.

The damage inflicted on the monolayer for different normal forces can still be differentiated for PS-based samples, so the point of absolute destruction has not been crossed. This means that the effect of the load regime on the monolayer damage can be studied. Factors that influence the severity of the load regime are certainly load, the underlying substrate parameters and relative velocity. As the velocity is the same in all experiments, the theoretical maximum Hertzian pressure between the probe and the substrate without HUVEC p_0_ may be a good indicator for the severity of the load condition, as it captures both load and material parameters. Also, pressure is an intuitive gauge of how damaging a load condition may be. In figure 16, this is visualized. Two datasets are plotted: the removed nuclei data for PDMS-based samples on the left and the equivalent for PS-based samples on the right. Both sets of data are plotted over the Hertzian contact pressure on the *x*-axis. The calculations were made for the respective substrate and an *r* = 1 mm glass probe. Note that the *x*-axis is in log scale, and there is a gap of two orders of magnitude between the two subplots. PDMS-based and PS-based experiments stand for mild- and harsh-load regimes, respectively, as indicated per the Hertzian pressure p_0_. When putting those two load regimes side by side, the bigger picture seems to become more apparent. For a mild-load regime, the cells are capable of remaining on the substrate to a large extent even if some die, as evident from the damage data and microscope images. However, the damage inflicted on the monolayer stays around zero and then rises relatively steeply towards the end of the mild-load regime. On the other hand, for the harsher-load conditions, the damage rises steeply but then seems to converge towards 1 (full destruction of the monolayer). Put together, the graphs look like they form the ends of a saturation curve, albeit the transition area between those ends is not depicted, and there is a large gap in between. Qualitatively, this makes sense, as for mild loads, few cells are being removed, while for harsh loads, nearly all are. Quantitatively, the large gap indicates that Hertzian pressure may not be the best measure for load regime severity. While the damage may be compared, the friction data may not because, as laid out earlier, it seems likely that the probe is considerably interacting with the underlying substrate as the monolayer is breached to a large extent for all cases tested on polystyrene.

## 6. Conclusions

In this article, a novel methodology was presented that can enable researchers to conduct ultra-low friction experiments under physiologically relevant conditions. Moreover, the presented protocols represent an affordable alternative to expensive microtribometers for groups that have access to a generic tribometer that can be used with low-load cells. Without soft substrate samples, however, the experimental set-up, as used in this work, is considered unable to test friction on cells cultured on polystyrene-based samples. Instead, the load conditions are so harsh that the monolayer is destroyed to a large extent, and consequently, a polystyrene substrate was tested against glass with the cell matter as a lubricant. This is evident from several observations. First, the majority of the monolayer was destroyed. As a large portion (46.3–80.3%) of cells do not remain in the track area, the probe has clearly penetrated the monolayer and must hence be interacting with the polystyrene to a large extent. As most cells are destroyed and the friction force in the dynamic case is significantly reduced, this implies that the remains of the cells are acting as some sort of lubricant. However, the friction forces are not reduced to a point where they would be comparable to literature values, or those collected on soft substrate samples. Overall, the load condition is simply too harsh for enough cells to survive in the track, which prevents testing the desired material pairing (cells against probe material). Experiments could still be conducted with different probe materials on PS samples; however, any differences in friction resulting from cell–probe interaction could not be easily differentiated from the ones resulting from cell-matter-lubricated PS–probe interaction. To ensure testing of the desired material pairing, a different, much more expensive, set-up would be required. However, differences in terms of damage caused to the monolayer were still measurable on PS samples and made sense when compared with the data collected on soft substrate samples, considering the harsher-load regime. It should be noted, however, that the indicator used to quantify the severity of the load condition here (p_0_) does not give credit to all factors that may influence the harshness for the cells and is thus almost guaranteed to not cover all cases of load conditions. This is evident from the large gap that extends over two orders of magnitude in figure 16. Furthermore, the friction data are not directly comparable between PS-based and PDMS-based samples as testing does not take place in the same load regime despite the same normal forces being used. The load cell that was used in this experiment was almost pushed to its upper limit by testing a normal force of 80 mN (the limit is 100 mN). To obtain a more continuous version of the plot and hence gain a broader view of the load regimes, soft substrate samples and the same type of probe could be used in conjunction with load cells capable of exerting higher loads. This would allow collecting data to cover the transition area and compare friction data over the whole range of load regimes, although still, at high loads, the substrate may be tested rather than the cells. For indentation, average Young’s modulus of vessel tissue is 125 kPa [12]. Clearly, the substrate used here is softer than this; however, compared to previous studies, the sample used here is closer to reality in terms of mechanical properties. In a future study, the PDMS could be adjusted to match physiological properties even better. The results of this work imply that tests conducted on monolayers cultured on hard substrates lack significance in terms of measuring damage and friction. This is because pressures are unrealistically high compared with practical applications, which destroys the monolayer and results in testing of the underlying material and not the cells. This means that in order to identify useful materials that could decrease friction and damage and in practice allow for less invasive interventions, soft substrates should be used. The probe images suggest that cells adhere to the probe. If this adhesion becomes too strong, the cells will be destroyed. Therefore, for practical applications, materials should be identified that exhibit low adhesive interactions with cells.

Misalignments within the friction testing set-up and unevenness of samples can cause significant deviations in the measured friction. The smaller the friction coefficient, the higher the influence of misalignments. While this effect has been accounted for in reciprocating experiments for dynamic friction, this work presents a new approach that also works for static and dynamic frictions of single-slide experiments. The soft substrate samples have proven an extremely affordable alternative to expensive microtribometers when used in conjunction with the approach to account for misalignments and unevenness. In the future, the methodology presented here may be used to conduct experiments using different probe materials and a range of load conditions to simulate more relevant *in vivo* conditions without the requirement for expensive equipment.

## Figures and Tables

**Figure 17.**
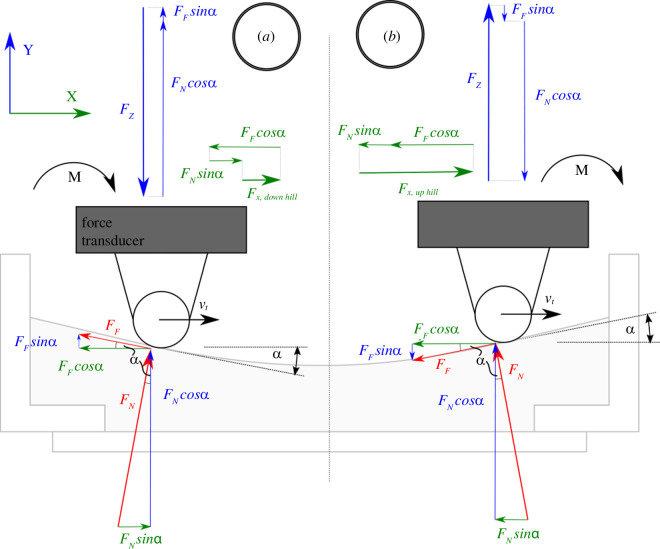
Free body diagram of forces acting on the probe and registered by the force transducer at either side of the sample.

**Figure 18.**
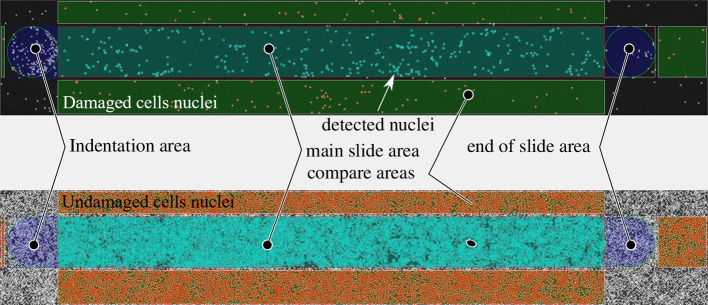
Red channel (dead, top) and blue channel (live, bottom) nuclei data with reference areas in green and orange, respectively.

**Figure 19.**
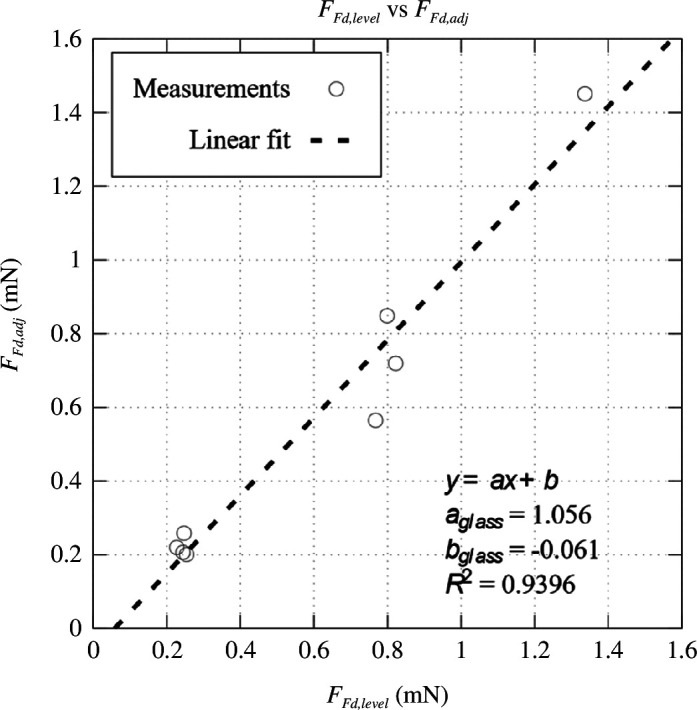
Zero-slope plotted against slope-adjusted dynamic friction forces.

**Figure 20.**
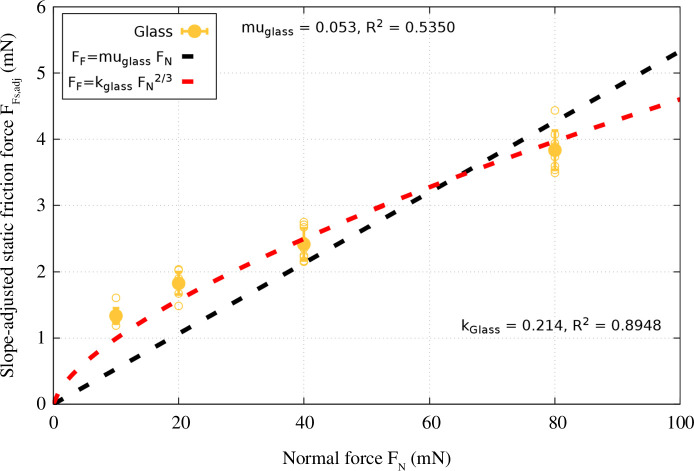
Slope-adjusted static friction force $F_{Fs,adj}$ on PDMS-based samples with HUVECs against glass probes plotted over applied normal force $F_{N}$ . Individual measurements are plotted as hollow symbols, while averages are indicated by filled ones with standard deviations. Amonton’s law fits $F_{F}$ = ${\mu F}_{N}$ (in black) and a Hertzian contact area-based fit $F_{F}=kF_{N}^{2/3}$ (in red).

**Figure 21.**
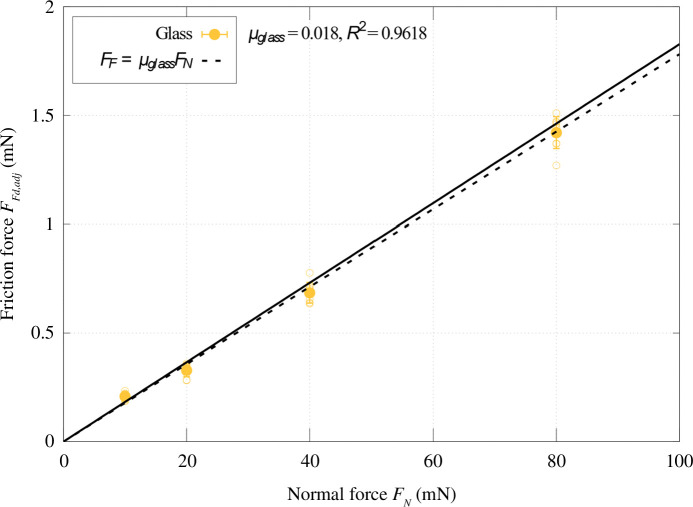
Slope-adjusted dynamic friction force over the whole range of applied $F_{N}$ fitted with Amonton’s law.

**Figure 22.**
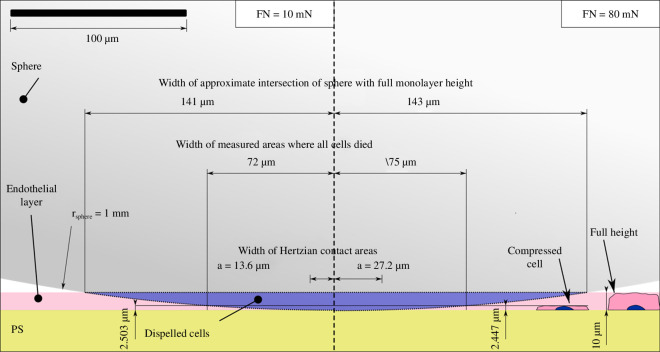
Contact geometry approximation between cells seeded on PS and a 1 mm radius glass sphere for a 10 mN (left) and an 80 mN (right) interaction drawn to scale. The scale is shown in the top left corner. A schematic cell layer height of 10 µm is annotated in addition to some important distances to the centre line. These include the calculated radius of the Hertzian contact area, the experimentally determined distance in which all cells died and the distances to the centre line where the gap size (distance between the sphere and the PS surface) is equal to the half and the full cell monolayer height. For the distance below which all cells died, the respective gap size is annotated.

**Figure 23.**
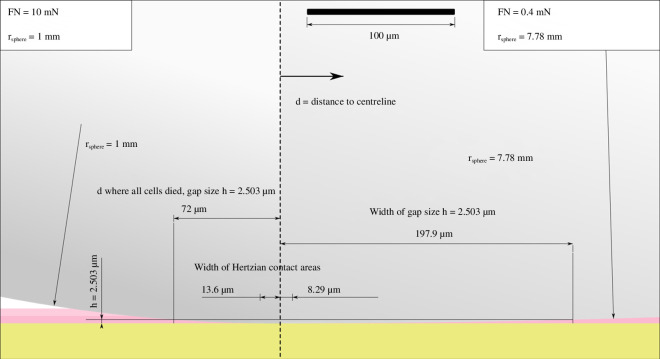
Hertzian contact geometry approximation of a 10 mN interaction with a 1 mm radius sphere compared to an experiment carried out by Dunn *et al*. [5] with a 7.78 mm glass sphere under a normal load of 0.4 mN. The figure follows the same notation style as in figure 22 and is also drawn to scale for both sides. The seemingly crucial gap distance of 2.5 µm was transferred to the approximation of Dunn’s experiment and the respective distance to the centre line of 197.9 µm is annotated.

**Figure 24.**
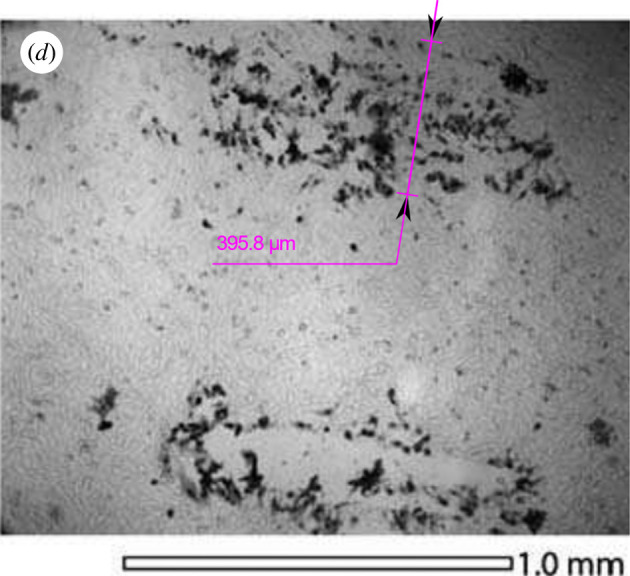
Trypan blue-stained monolayer of bovine aortic ECs after friction testing. Adapted from Dunn *et al*. [5]. The annotation, marking a distance of 395.8 µm in pink, was added.

## Data Availability

Data are available from Zenodo [13,14].

## Appendix A. Approaching misalignments of sample

The issue of misalignment is a known problem of low-friction experiments [15–17]. The probe moves from left to right over the sample with speed $v_{t}$ under set applied load $F_{z}$ while the force in the *x* direction $F_{x}$ is measured. Under relative movement between the sample and the probe, contact force $F_{N}$ causes friction force $F_{F}$ at the interface, which is acting against the movement, parallel to the sample surface. If the sample surface was perfectly aligned with the force transducer and the direction of movement, the friction force would not have a vertical component and, hence, $F_{x}=F_{F}$ . However, in the real world, the sample surface will not be perfectly aligned with the force transducers, and therefore the contact force between the sample and the probe may not only act in vertical direction. This results in a horizontal component of the contact force, which will be detected by the load cell in the *x* direction. As the sum of all forces in the horizontal direction is measured as $F_{x}$ , the horizontal component of the contact force can have an impact on the measured friction force. For most traditional applications, this effect may be negligible as friction coefficients are usually much larger. In the case of cell biotribology, however, friction coefficients of around 0.03–0.06 [5] can be expected. For a sphere pressing on a surface with force $F_{y}$ under misalignment angle α , the relation between $F_{y}$ and the resulting force in the *x* direction $F_{x}$ is $F_{x}/F_{y}=\tan\left(\alpha \right)\approx \alpha$ . Hence, to obtain a reaction force in the *x* direction that would exceed the expected friction force, only a misalignment angle α of $0.03\;rad=1.72^{\circ }$ or 30 µm height difference on 1 mm of sliding distance to 0.06 rad ( 3.44° ) is necessary. Such a misalignment would cause the measured friction force to either completely cancel out or double in value, depending on the direction of misalignment. The misalignment necessary to significantly interfere with the measured friction force is much smaller.

In previous studies [15–17], the problem was solved by combining the measurements of two slides on the same track, one on the way forward and one again on the way back. However, the experiments carried out in the scope of this work only involved a slide in one direction. Even for multi-slide experiments, this approach comes with three major downsides that disqualified it for application in this work. First, as with all tribological experiments, the interacting surfaces may change throughout the course of the experiment. For cell monolayers, this is even more of an issue than for traditional mechanical engineering materials because the cell layer may be damaged or destroyed in parts, or even be fully removed. Second, the approach makes it hard to measure the static friction coefficient as it (except during stick–slip) only occurs at the beginning of a slide. Hence, on the backwards slide, at the end (returning to where the forward slide began), only dynamic friction occurs making it impossible to average static friction. Third, as the majority of this work was carried out using a soft substrate that may deform considerably owing to shear, it cannot be ensured that positions are matched up exactly. Only the stage position can be measured; however, the respective position on the sample, and with it the slope, may be different on the way forward and on the way back for a single given stage position.

For these reasons, an alternative and novel approach to account for the issues of measuring low friction was developed in this work. In figure 17, the forces acting on the probe and the ones measured by the force transducer are shown. As the probe moves downhill on the sample, as shown in figure 17, the respective measured force in the *x* direction $F_{x,downhill}$ is reduced by the *x* component of the normal force between sample and probe $F_{N}\sin\alpha$ and only the *x* component of the friction force $F_{F}\cos\alpha$ is measured:

(A 1)
$$F_{x,downhill}=F_{F}\cos\alpha -F_{N}\sin\alpha .$$

If the probe moves uphill on the sample, as shown in figure 17, $F_{x,uphill}$ is increased by $F_{N}\sin\alpha$ :

(A 2)
$$F_{x,uphill}=F_{F}\cos\alpha +F_{N}\sin\alpha .$$

The measured force in the *z* direction $F_{Z}$ is influenced by this effect too; however, as both the angle and the friction force are usually small, the effect on $F_{Z}$ is very small, too.

To account for this, the following method was developed: when the desired load has been applied, but the stage has not moved yet and is stationary, the friction force is consequently 0. Accordingly, as per the mechanical model introduced in figure 17, the forces measured by the load cell consist entirely of the ones exerted on the probe by the substrate. When the stage is then moved, this information can be used to adjust for the slope. This is shown in figure 6. The value for the force measured during the loading phase a (red) is used as offset when calculating the static friction force measured during position *b* (green). The same approach can reduce the slope’s effect on the dynamic friction force. In practice, there remains a residual influence. This is because the dynamic friction force takes a while to stabilize (*c*, blue box). A better way to measure the dynamic friction force is to measure it at the area where the slope is around zero. To find this area, the height of the probe $h_{m}\left(x\right)$ was approximated with a fifth-order polynomial $h_{p}\left(x\right)$ . The derivative of $h_{p}\left(x\right)$ in the *x* direction $h_{p}^{`}\left(x\right)={\partial_{x}h}_{p}\left(x\right)$ is the approximate slope of the sample. This can be used to find the location where the surface is horizontal, as shown in figure 6 (*d*, grey). However, the slope never reaches zero for some slides owing to substantial misalignment. Hence, where possible, three values were calculated: the slope-adjusted static friction force $F_{Fs,adj}$ , the slope-adjusted dynamic friction force $F_{Fd,adj}$ and the zero-slope dynamic friction force $F_{Fd,level}$ .

## Appendix B. Determining monolayer damage

The programs that were used are CellProfiler (open source) [18] and MATLAB. Blue and red images of the whole testing site on the stained sample are required. For a 10 mm slide, 6 and 7 images at 4× magnification were collected and captured with a Nikon Eclipse Ti. The microscope writes *x*, *y* and *z* positions into the metadata of the images. Lengths were calibrated to convert from pixels to mm.

Using the stage positions, the images were stitched together to cover the whole track. Areas surrounding the slide track were captured for each slide to allow for a reference area. These stitched blue and red channel images were then loaded into CellProfiler to detect the positions of the nuclei which were then saved to two separate csv files. The respective slide and reference areas were then determined manually. In figure 18 , the data for damaged and live cell nuclei are shown with respective reference areas in green and orange.

These data were used to calculate the average nuclei density ε in nuclei per pixels:

(B 1)
$$\varepsilon =\frac{n}{A^{\prime }},$$

with the number of nuclei n and area in pixels A` . To obtain the average nuclei density in nuclei mm^−2^, area A` must be converted to mm^2^ with a calibration factor ξ that describes the relation of lengths in pixels to their respective length in mm^2^:

(B 2)
$$\rho =\frac{n}{\xi^{2}A^{\prime }}.$$

Furthermore, the relative nuclei density in the slide area $P_{slide}$ is calculated and reflects the effect of frictional interaction on nuclei density relative to the reference areas:

(B 3)
$$P_{slide}=\frac{\rho_{slide}}{\rho_{compare}},$$

with $\rho_{slide}$ being the average nuclei density in the slide area and $\rho_{compare}$ being the respective counterpart in the reference areas. The amount of removed cell nuclei was calculated through

(B 4)
$$P_{removed}=1-\frac{P_{slide,blue}+P_{slide,red}}{P_{compare,blue}+P_{compare,red}}.$$

## Appendix C. Friction measurements

In order to study the correlation of $F_{Fd,adj}$ and $F_{Fd,level}$ on soft substrate samples, the two forces are plotted against each other in figure 19. By plotting individual slides’ measurements with the zero-slope dynamic friction force $F_{Fd,level}$ on the *x*-axis, and the slope-adjusted counterpart $F_{Fd,adj}$ on the *y*-axis, along with a linear fit of the form *y* = *ax* + *b*, the correlation between the two was assessed. Ideally, the two should match up perfectly, resulting in a line with a slope of 1 that intersects the *y*-axis in the origin. The slope *a* was 1.056 and the line intersected the *y*-axis at *b* = −0.061. This indicates that there is a correlation between the two values, as is expected. Assuming that $F_{Fd,level}$ is the acurate dynamic friction force, *a* being consistently larger than 1 indicates that $F_{Fd,adj}$ is consistently overestimating the dynamic friction. The reason for this is likely that the dynamic friction force can only be measured once it has stabilized. Therefore, as long as the sample is not perfectly flat, there will be a difference in the slope between the location where the initial slope influence was measured and the location on the slide where the dynamic friction force has stabilized and is measured. Accounting for the overestimation when using $F_{Fd,adj}$ is difficult and would require an advanced model. However, from the fitting data, it can be deduced that the implications of this effect are of the order of 5.6% overestimation of the friction force.

The static, slope-adjusted friction forces $F_{Fs,adj}$ are plotted over $F_{N}$ in figure 20 with individual measurements and averages. The friction coefficient was fitted with Amonton’s law, with friction coefficients *µ* of 0.053. However, the fit does not correlate very well according to the coefficient of determination.

The data for the static friction force were also fitted with another function under the assumption that the static friction regime is heavily adhesion-dominated and could, therefore, be dependent on the (apparent) contact area. Furthermore, the assumption was made that the adhesive stress is load-independent. According to the Hertzian contact theory for a sphere and a flat surface, the contact area radius *a* is related to the normal load $P_{0}$ , which here is $F_{N}$ , by $a\propto F_{N}^{1/3}$ . This Hertzian approach does not account for adhesive effects; however, also, in the JKR model, *a* is largely proportionally related to $F_{N}^{1/3}$ . Therefore, the contact area $A_{H}=\pi a^{2}$ is assumed proportionally related to $F_{N}^{2/3}$ and hence $F_{F}=kF_{N}^{2/3}$ with fitting factor k . The model fits the static friction data much better than Amonton’s law with the respective $R^{2}$ of 0.8948. This could mean that indeed the interaction could be mainly owing to adhesive connections, for example, in the form of chemical bonds between probe and cells.

In figure 21, the slope-adjusted dynamic friction forces are plotted over $F_{N}$ in the same way. Overall, the friction forces are much smaller than on hard samples, and it should be noted that a fit with Amonton’s law seems to correlate well with these data. Hence, Amonton’s law predicts dynamic friction far more accurately for this application than it does for static friction. The dynamic friction coefficient $\mu_{d,glass}$ was very small with a value of 0.018.

## Appendix D. Contact width study

Assuming a largely Hertzian-dominated contact interaction, and cells being so soft and fragile that they play no role in the interaction from a mechanical point of view, giving way to the probe material when there is any contact, the following would be the case.

The interaction is drawn to scale in figure 22. A cross-section of a Hertzian indentation is shown between a glass sphere of radius $r_{sphere}$ = 1 mm and the polystyrene dish as was tested in those experiments. In between those materials, HUVECs were trapped in the interaction. However, owing to their low Young’s modulus (reported values range from 400 Pa [19] to 1 MPa [20], depending on the measurement location) compared with PS (3.25 GPa), their influence on the contact geometry can be neglected which is hence only determined by the glass sphere and the PS dish. ECs are generally between 0.1 and 10 µm thick [21,22]. The full height of an EC monolayer (10 µm) is shown in the figure with a full-height cell. On the left, an indentation is shown with a load of 10 mN and on the right-hand side there is one with 80 mN. Several distances from the centreline are annotated. The Hertzian contact radius *a* is shown for both cases. Additionally, the estimated contact width radii from figures 7 and 8 are annotated for 10 and 80 mN, respectively. Furthermore, the maximum distances are shown at which sphere and polystyrene surfaces are 10 µm away from each other. These locations constitute limitations as to the distance to the centreline where the sphere could still be in physical contact with cells. From this model, it is obvious that even though the sphere and dish are not in immediate contact with each other beyond the Hertzian contact radius, the distance between them (also referred to as gap/gap size from here) may be too small to allow a cell to survive in the gap between them as it would simply get squashed. Such a cell would be damaged or partially or completely removed from the surface as the sphere moves along it (once relative movement is introduced). By annotating the measured width of the contact, which was extracted from the microscope images, the respective gap size was determined. For 10 mN, this is 2.503 µm, and for 80 mN of normal force, it is 2.447 µm. Next to the cell on the right, a cell that was squashed to that height is shown. As the measured values are very close together, this supports the thesis that the size of the gap between the two surfaces is crucial for the survival chances of a cell that is trapped in it. This model has some limitations. As already mentioned, it still only considers the most basic of contact mechanics, which is not an accurate representation of the contact (especially once the sphere and the dish move relative to each other) owing to adhesive effects, relative movement of the interacting bodies, etc. A Hertzian calculation of the contact, however, should still give a good estimate of the contact width. Furthermore, the model disregards deformations of the cells. While this may not have any significant mechanical implications, it leads to the monolayer geometry not being represented accurately. As the cells must be squashed outwards, they may bulge along the sphere, possibly deforming the monolayer around them which could lead to more cells being in contact with the sphere. Also, a cell monolayer is not flat, but it has junctions and varies in height [23]. However, the 10 µm line represents a gauge for the maximum height of the monolayer. Furthermore, a pure interaction (with a low pressure) between probe and cell is not necessarily deadly for the latter, neither is it likely to remove the cell as was shown with the experiments on PDMS-based substrates.

Overall, despite its limitations, the model can explain some findings. First, the observation that the experimentally determined track width is greater than the predicted Hertzian contact width can be attributed to the fact that, even outside of the contact area, the distance between the sphere and the PS dish is too small for a cell to fit in. This can lead to cells being squashed and removed or standing a good chance of remaining unharmed, depending on their height and distance to the centreline. This also explains why there are variances in the track width along the slide where some cells may be dead but are often not removed. Those cells were likely too big to fit between the sphere and the dish and hence got destroyed but did not quite have enough contact with the sphere to remove the cell remains from the PS. Finally, this model also explains why, despite increasing the Hertzian contact area with an increase in normal force, the experimentally determined track width does not increase to the same extent. This is because it seems the survival of a cell is strongly determined by the size of the gap between the sphere and dish surfaces on which the Hertzian contact radius only has a limited influence. The experiments on PDMS have shown that a simple interaction between sphere and cell is likely not deadly for glass probes. Therefore, the survival chances of a cell that is trapped in the gap between the sphere and the dish depend largely on the cell size and its distance to the centreline. Hence, it is unlikely to find cells that were killed by the interaction past a certain distance because apart from the ones that are too big, they can deform and survive. On the other hand, there is a zone within a certain distance to the centreline, in which almost all cells are removed and only very few survive healthily, owing to the small gap size. The latter distance can be gauged to around 72–75 µm with a gap distance of around 2.5 µm. This is slightly dependent on the load condition and highly dependent on the sphere diameter. It is not immediately clear why the critical gap distance was 2.5 µm.

Peeters *et al*. studied the failure properties of single mouse myoblasts. They compressed attached cells with a flat indenter and studied reaction forces and strains. The authors report cell bursting to occur at a load of 8.7 ± 2.5 µN at a strain of 72 ± 4% [24]. Assuming similar mechanical and failure properties for HUVECs, a cell’s maximum height at the failure point can be estimated with 10 µm × (100–72%) = 2.8 µm. Thus, the observation of 2.5 µm being the size of the critical gap agrees well with the findings of Peeters *et al*. [24].

The implications of this model should have also affected previous studies. Dunn *et al*. [5] have conducted experiments with a different type of cells (bovine aortic ECs) that were cultured on cell culture grade PS, which is the same type of material used in the experiments here. The monolayers were tested against a borosilicate glass probe of radius 7.78 mm under normal loads of 0.4–1.2 mN [5]. For reference, the probes used here were made from soda lime glass and of 1 mm radius. Also, the normal load in the experiments here was much higher at 10–80 mN. A schematic of the contact geometry for the experiment of Dunn *et al*. is shown in figure 23 on the right side for a normal force of 0.4 mN against an experiment conducted in the context of this work for 10 mN on the left. Both sides are drawn to scale. From the experiment here, the width of the track was estimated to a radius of 72 µm with a respective gap size *h* of 2.503 µm. Transferring this gap size to the experiment of Dunn *et al*. in a contact geometry drawn to scale for their experiment conditions results in a distance to the centreline of 197.9 µm as shown in figure 23.

Dunn *et al*. included images of the post-friction testing, trypan blue-stained monolayer in their publication. One of their images is adapted in figure 24. In addition to their microscope picture, in the figure, a distance of 2 × 197.9 µm = 395.8 µm is marked. In the case of their experiments, this distance seems to give a good estimate of the width in which cell death was found; however, in this particular experiment, not many cells were removed. It should be noted that the authors achieve very low pressures with their testing methodology both by applying forces which are orders of magnitude lower than the ones used in this work and also by using a sphere of a radius that is much larger than the one used here as a countersurface. These low pressures likely lead to reduced damage to the monolayer and the lack of cell death that is observed in this specific experiment. Dunn *et al*. computed the occurring pressures to be in the range of 3–5 kPa with significant damage to the cell monolayer occurring from 5 kPa [5]. The dimensions of an EC are 50–70 µm in length and 10–30 µm in width [22]. Knowing these dimensions, one cell covers an area of around 500–2100 µm^2^. The critical pressure at which cells burst and are thus destroyed can be estimated now with that area and the finding of Peeters *et al*. that a load of 8.7 ± 2.5 µN results in a cell bursting. The estimated bursting pressure is in the range of 4.143–17.4 kPa. Even though this is only an estimate, the resulting pressure is of the same order of magnitude as the calculation of Dunn *et al*. Estimating the contact pressure from the measured contact width (like Dunn *et al*. did) for the experiments conducted here, yields a pressure of 614–4527 kPa, for normal forces of 10 mN to 80 mN with contact radii of 72 and 75 µm, respectively. This calculation was made assuming a circular-shaped contact area. The pressures occurring in the experiments here are much larger than the ones Dunn *et al*. [5] report to cause significant damage. They are also much larger than the estimated burst pressure calculated with the results of Peeters *et al*. Therefore, it is not surprising that in the experiments here almost all cells in the affected area were completely destroyed.

In conclusion, the observations that the contact area width is larger than Hertz predicts, and that it only changes slightly when increasing the normal force, make sense in the context of the model. While the model has its limitations, it agrees with the findings of previous studies and qualitatively explains the observations made when testing a monolayer cultured on PS against a glass probe. The model validity was confirmed when applying it to the results of Dunn *et al.* figure 23 .
